# The Influence of Some Physicochemical Parameters of Surface Waters on the Formation of Trihalomethanes During the Drinking Water Treatment Process

**DOI:** 10.3390/molecules30142983

**Published:** 2025-07-16

**Authors:** Alexandra Scarlat (Matei), Cristina Modrogan, Magdalena Bosomoiu, Oanamari Daniela Orbuleț

**Affiliations:** Department of Analytical Chemistry and Environmental Engineering, Faculty of Chemical Engineering and Biotechnologies, National University of Science and Technology Politehnica Bucharest, 7 Polizu Street, 011061 Bucharest, Romania; alexandramatei974@yahoo.com (A.S.); magdalena.bosomoiu@upb.ro (M.B.); oanamari.orbulet@upb.ro (O.D.O.)

**Keywords:** trihalomethanes, drinking water, pH, temperature, amount of natural organic substances in water

## Abstract

Trihalomethanes (THMs) are a class of disinfectant by-products present in chlorinated tap water. Mainly due to their carcinogenic potential, their concentration in drinking water is now limited by regulations. In Romania, little is known about their distribution in urban drinking water supply systems, their magnitude, or their seasonal variation. Drinking water suppliers periodically adapt and optimise their water treatment methods for economic reasons and in response to regulatory changes and technological developments. The formation of THMs is influenced by the physicochemical parameters of water (pH, temperature, total organic carbon—TOC) and by environmental factors (geographical, climatological). Most of these factors have significant seasonal variations that lead to the formation of THMs in variable concentrations. In this study, we analysed the seasonal trends in surface water quality (considering variations in temperature, pH, and TOC) and correlated them with the concentration of THMs in drinking water over two calendar years. Water samples were collected from the Arges River, in a geographical area comprised of plains. The results show that the formation of THMs is enhanced by increasing temperature over the course of a year, with the highest concentrations being obtained in July 2022 (98.7 µg/L THMs at 30.5 °C) and in August 2023 (81.9 µg/L THMs at 30.4 °C). The main parameters that trigger the formation of THMs are the organic matter content and the disinfectant dose; the pH has a moderate effect, and its effect is correlated with the concentration of organic matter. There were noted strong seasonal changes in the concentration of THMs, with the maximum peak being in the middle and late summer and the minimum peak being in winter. This indicates the possibility that the quality of drinking water may change as a result of climate change. In addition, monitoring and chlorination experiments have established that the concentration of THMs is directly proportional with the TOC.

## 1. Introduction

The prevalence and severity of some diseases are linked to a lack of sufficient and clean water. The degradation, quantitative limitation, and lack of accessibility of water sources means that the water treatment process should also use more contaminated sources which requires the application of advanced, more expensive technological methods to produce clean water. Waterborne diseases are caused by pathogens (e.g., responsible for dysentery, diarrhoea, giardiasis, *E. coli* infection, typhoid fever, salmonellosis) that are transmitted to humans through contact with or the consumption of contaminated water [1]. The microbiological surveillance of drinking water has been practiced since the beginning of the 20th century to prevent water-borne diseases. The possibility of water contamination with pathogens in water distribution systems (e.g., during network maintenance work), natural events (e.g., floodings), and deficiencies in the water treatment process are known as causes of water-borne epidemics. The most important step in making water suitable for human consumption, to avoid the risk of transmitting infectious diseases of an epidemic nature, is disinfection. Although there are several methods that are currently studied (e.g., use of photothermal materials [2], ion-exchange [3], photocatalytic and photo-electrocatalytic processes [4], piezoelectric processes [5], solar water disinfection [6]), chlorination is still the most used method for water disinfection, because it offers the advantages of being cheap, efficient, and relatively easy to implement [7,8,9]. Also, compared to other methods, chlorination is efficient in preventing subsequent water contamination during transport and biofilm formation in water distribution systems [10].

Disinfection by-products (DBPs) are made up of two major classes: trihalomethanes (THMs) and haloacetic acids (HAA5 which include monochloroacetic acid, dichloroacetic acid, trichloroacetic acid, monobromoacetic acid, and dibromoacetic acid). They are usually formed when water is disinfected by chlorination or ozonation: the disinfection agent reacts with chemicals present in the water to form DBPs [11,12]. The organic compounds that are present in surface water are naturally occurring chemicals and contaminating substances (originating from industrial, agricultural, and urban activities). The presence of complex organic compounds and treatment by-products in water generates chemical mixtures that can pose a health risk, especially since knowledge of their effects is still limited [13]. From the perspective of the normative in force, the health risk that disinfection by-products pose is considered small compared to the risks associated with inadequate disinfection; however, recent studies have underlined that even in small traces, (ppb levels) they pose a health risk [14]. The maximum allowed values of THMs (calculated as the sum of chloroform, bromoform, dibromochloromethane, and bromodichloromethane) in drinking water recommended by the European Union is 100 μg/L [15], that of the United States Environmental Protection Agency (USEPA) is 80 μg/L [16], and that of the WHO World Health Organization (WHO) is 200 μg/L [17]. While the USEPA’s recommendation is the strictest, the WHO’s recommendation is the oldest (1993); the EU guideline advises that the operators of drinking water facilities should strive to achieve lower values whenever possible. The WHO also has a recommendation for bromoform, which must be below 60 μg/L.

DBPs are formed during the water treatment process, but also in water distribution systems. When adding excess disinfectants to keep water microbiologically safe, the disinfectant reacts with organic substances in the water or in the biofilm formed on the distribution pipes [11]. The formation of THMs is a complex process that is enhanced by the presence of solids and chemical contaminants in the surface water. If these contaminants remain in traces in the treated water, they will act as THM generators. It has been demonstrated that manganese present in drinking water can undergo further oxidation and accumulate on the plastic pipes of drinking water supply systems. The manganese layer can act as a deposition site for organic compounds that are precursors to the formation of THMs [18]. It was found that biodegradable microplastics (MPs) are THM precursors. They interact with chlorine during the disinfection process and generate THMs; the most reactive component of MPs that contributes to the formation of THMs is poly (lactic acid), PLA [19]. Also, MPs can adsorb THMs that can easily be later desorbed under the action of UV light [20]. Combining two disinfection methods can also lead to increased rates of the formation of THMs; UV/chlorine treatment methods of an ammonia-contaminated water source generated more THMs than chlorination alone, especially at a pH of 9 [21]. Previous studies have reported that the physicochemical parameters of the water (such as dissolved organic matter—DOM, temperature, and pH), contact time, and the type and dose of the disinfectant are the variables that mostly influence the formation of THMs [22,23,24]. Fang et al. (2023) found that an alkaline pH during the chlorination step of water with different DOM content reduces the content of THMs [25]. On the contrary, Sriboonnak et al. (2021) reported lower THM concentrations at a pH < 7, compared with a pH > 8 [26]. This suggests that the content of THMs is probably more influenced by the nature of the organic matter present in the water, which is more or less reactive depending on the pH values. A study performed in Ireland also identified geographical factors (agricultural land, peat soil rich in organic matter combined with prolonged rainfalls) that caused the highest levels of THMs among EU countries to be recorded [27]. Tropical peat water was also found to contain large amounts of dissolved organic matter that favours the formation of disinfection by-products [28]. Tannic acid is frequently present in both raw and drinking water, generated by the decomposition of organic matter which is contributing to the formations of THMs [29]. The population supplied with surface water is more exposed to THMs than the population using groundwater; this difference is a consequence of the lower organic content of groundwater sources [30].

There are three major routes of human exposure to THMs: ingestion, inhalation, and dermal contact. Chloroform and bromodichloromethane are classified as Group 2B carcinogens (possibly carcinogenic to humans), while dibromochloromethane and bromoform are classified as Group C (unrated as to its carcinogenicity to humans) [31]. Some researchers have reported a real risk of cancer development associated with all THM compounds most likely linked to long-term exposure and synergistic effects in the nitrate presence [32,33]. Although, the most devastating effects of THMs are caused by their carcinogenicity (colon, bladder, pancreas), THMs can also create damage to various internal organs (stomach, brain, lungs, or liver) and can have negative effects on the reproductive system (intrauterine growth retardation, preterm birth, congenital malformations) [34,35,36,37]. Among THMs, chloroform was found to be the most abundant in the urban drinking water supply system of Pakistan [37]. This was valid also for the composition of THMs in Addis Ababa’s drinking water [38].

This paper aimed to study the level of contamination of drinking water with secondary disinfection compounds in water supplied in urban areas in Romania. For that a drinking water treatment plant located in southern region of Romania was chosen; this plant uses disinfection with chlorine compounds, so the targeted contaminants are trihalomethanes. The water source is the Arges River, an important river that is used to supply water to a large population group. Seasonal variations in the parameters of temperature, pH, and TOC, monitored for two years (2022 and 2023), were linked with the concentration of THMs formed in the disinfection process. The identified correlations can help to establish the conditions that minimise THMs formation and to predict conditions for which peak concentrations of THMs would be recorded.

## 2. Results and Discussions

Before starting the experiments, we performed a characterisation of the studied surface water. The experimental results are presented in Table 1.

### 2.1. The Influence of Temperature and Disinfectant Dose on THMs Formation

**Figure 1:**
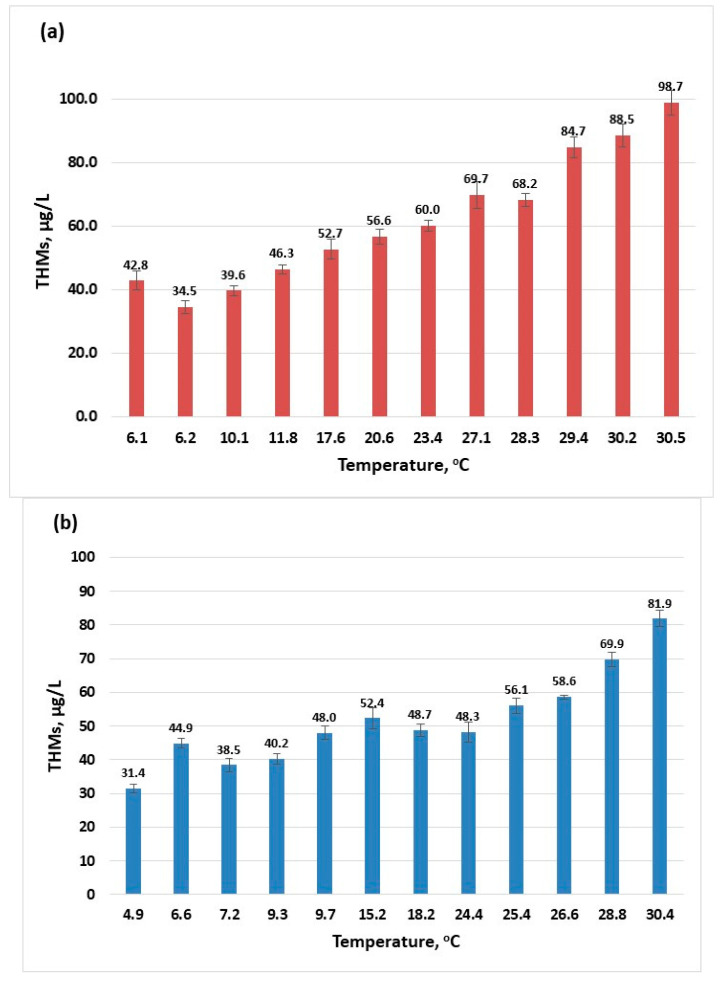
The influence of temperature on the formation of THMs (sampling 2): (**a**) 2022; (**b**) 2023.

The temperature is one parameter that influences the physical, chemical, and biological processes that occur during water treatment. According to the results given (Figure 1), the increase in temperature directly influences the formation of trihalomethanes. As the surface water temperature increases, the amount of THMs also increases. For the year 2022, the highest amount of THMs was 92.7 µg/L (Figure 1a) while in the year 2023, a maximum value of 81.88 µg/L THMs was recorded (Figure 1b). According to the European legislation, there are effluent discharges allowed (urban, agricultural, and industrial) into rivers if these discharges respect the conditions settled for the mixing zones [39]. Although the maximum recorded temperatures for the two years are close, there is a difference in the maximum concentration of THMs that can be attributed to changes caused in the river composition from one year to the other by these authorised discharges.

**Figure 2:**
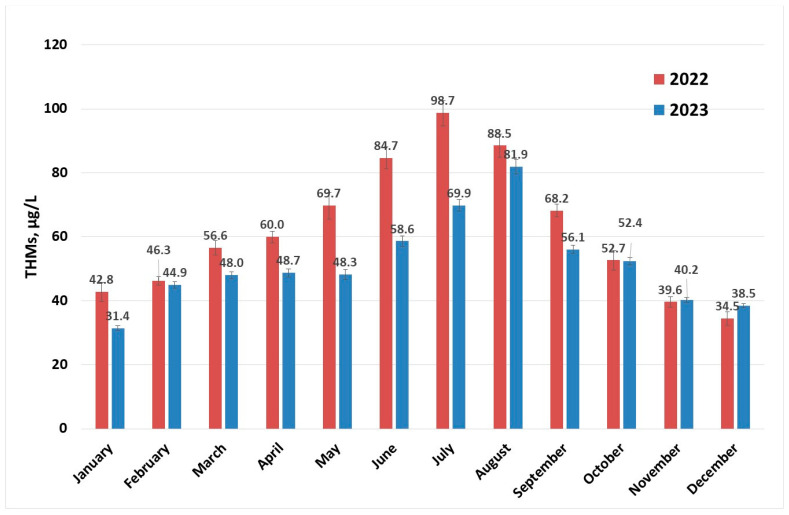
THM (sampling 2) concentration evolution per year.

Determinations of levels of THMs were carried out throughout the year for each month at different surface water temperatures. The concentration profiles of THMs after the disinfection process (described in Section 3.2) for the years 2022 and 2023 are presented in Figure 2. There is a clear increase in the content of THMs in the summer months; some of these values are close to the maximum recommended value by the European normative [15] and for July 2022, the 100 µg/L limit is surpassed. This indicates that if the conditions in the water supply system are met, the maximum value for THMs specified in European normative can be exceeded [30,40,41]. Values in the range 0.2–122 µg/L were reported for drinking water collected from facilities located in France that were using chlorination [30], while a maximum concentration of 157 µg/L (over two years of sample collection and analysis) was recorded in Australia [41] and 189.52 µg/L in Thailand [26].

**Figure 3:**
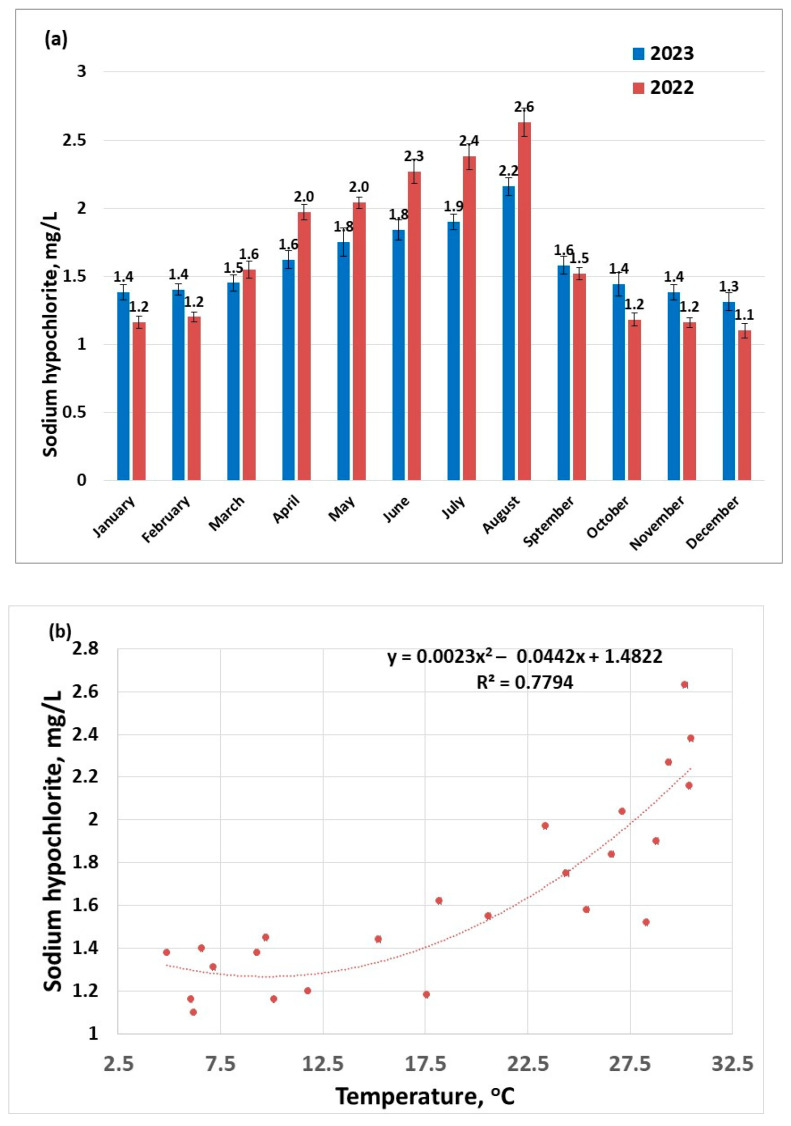
Disinfectant doses applied to raw water samples (sampling 2) for the years 2022–2023: (**a**) distribution per months; (**b**) distribution as a function of the temperature.

An increased dose of sodium hypochlorite is used to disinfect water during warmer months (Figure 3a), due to conditions that are conducive to the development of pathogens (e.g., thermotolerant pathogens like Legionella pneumophila, Naegleria Fowleri could proliferate) [42]. Increasing water temperature was also found to enhance the activity of ammonia-oxidizing bacteria [43]. Another explanation for choosing to use an increased disinfectant dose during warmer seasons is that the disinfectant is volatile; the higher the temperature, the more disinfectant will desorb from the water; this statement is supported by the nonlinear dependence of disinfectant dose on temperature (Figure 3b). This suggests that during warmer seasons, it is more difficult to maintain a safe dose of excess disinfectant to guarantee a pathogen-free drinking water.

**Figure 4:**
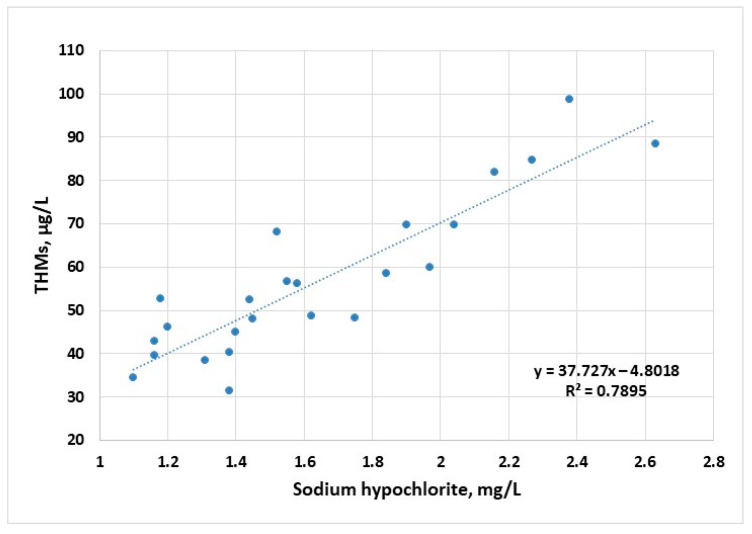
Evolution of THM concentration (sampling 2) as a function of disinfectant dose applied to raw water samples for the years 2022–2023.

The corresponding disinfectant doses applied to raw water samples follow the profile of the maximum concentrations of THMs, suggesting a strong connection between THM concentrations and the available chlorine ions (Figure 4). First- and second-order polynomial correlation between disinfectant dose and the content of THMs have been previously reported [44].

One solution to keep the temperature in the distribution pipe under control (and therefore to limit the formation of THMs, the volatilisation of disinfectants, and the development of pathogens) is to install the water distribution system as low as possible underground. Studies have evidenced that in summer, the water temperature between the outlet of the drinking water treatment plant and the distribution system can reach differences of up to 8 °C, in the best case, for pipes buried at great depths [45]. With the increase in global temperatures, the occurrence of urban heat islands in cities will become more frequent and will record increasingly high maximums. This will put even greater pressure on the correct adjustment of the disinfectant dose.

### 2.2. The Influence of Total Organic Carbon (TOC) on the Formation of THMs

TOC is not specifically regulated in the European Union; however, there are specifications that this parameter should be monitored in the drinking water treatment plant for abnormal changes [15]. TOC is however linked with the presence of THMs in the drinking water because during the disinfection process, chlorine reacts with organic matter and leads to the formation of trihalomethanes. In this case, alternative guidelines are used; these have set the levels for TOC in raw water at 2.0 mg/L; whenever this value is not respected, supplementary indicators are introduced (e.g., TOC in raw water below 4.0 mg/L and THMs below 40 µg/L) [46].

**Figure 5:**
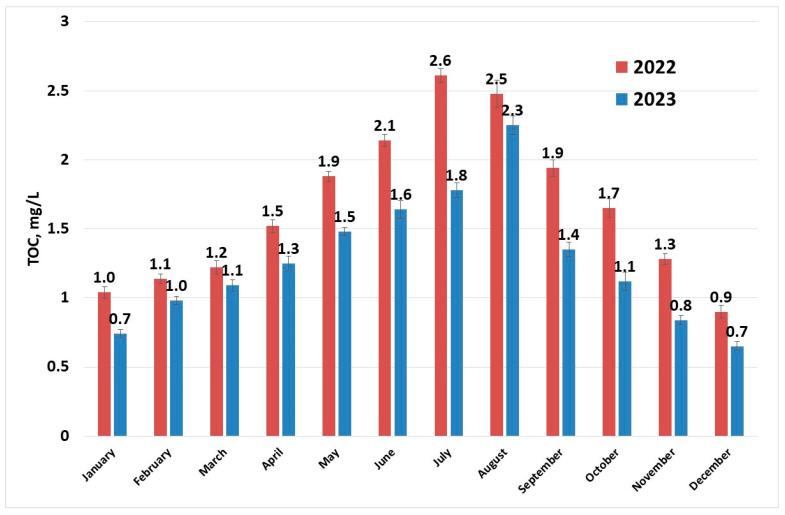
TOC (sampling 1) evolution in raw water per year.

Figure 5 shows that the maximum values of TOC occur during the summer season. Organic matter in rivers is mainly composed of humic substances [47]. Humic substances are residues resulted from decomposition of dead plants and microorganisms. They are present in soil sediments and water. Consequently, unless a major pollution event with organic substances comes to perturb this equilibrium, TOC values follow the temperature profiles.

**Figure 6:**
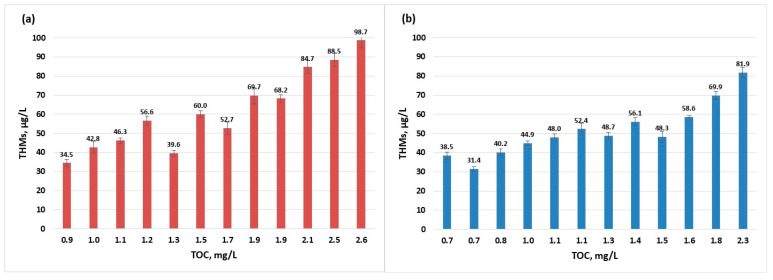
THMs (sampling 2) vs. TOC evolution in raw water per year: (**a**) 2022; (**b**) 2023.

During the summer months although the concentrations of THMs fall in the worst-case scenario just below the European limit (100 µg/L), the guidance value for TOC is not respected (TOC values below 2.0 mg/L) (Figure 6). When analysing and considering the next level of safety (TOC below 4.0 mg/L and THMs below 40 µg/L), the data from July to September 2022 and August 2023 did not comply with the advised limits. Extensive study on the removal of different organic compounds from water sources using several processes has shown that coagulation is efficient for the removal of high-molecular weight organics, adsorption on activated carbon is efficient for the elimination of low-molecular weight organics, electrodialysis reversal especially removes the aromatic compounds, while ion exchange removes the humic substances [22]. Among the above enumerated processes, the present treatment process is using coagulation and filtration, therefore the humic fraction, representing most of TOC constituents [47], is not efficiently removed.

**Figure 7:**
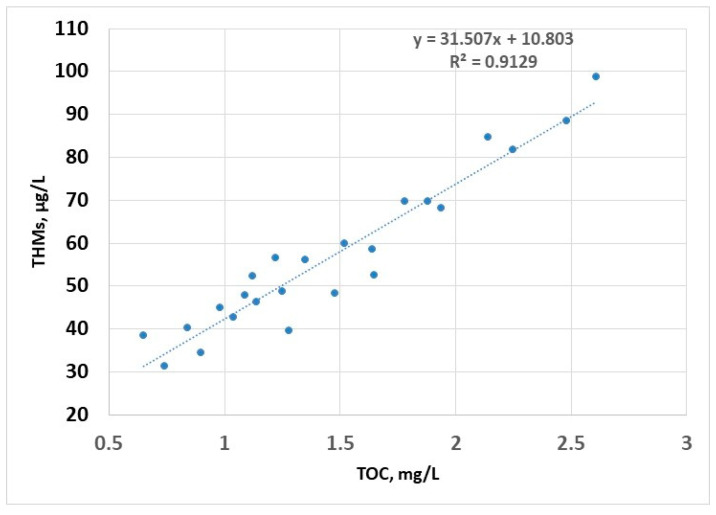
THMs (sampling 2) vs. TOC—linear regression.

The linear regression model shows a strong dependence of the content of THMs on TOC (R^2^ > 0.9); this is due to the fact that the disinfectant is always in excess (as a residual chlorine for the potential disinfection of water in the distribution system), so that the only limiting factor in the formation of THMs is the concentration of organic compounds; this model can be used to estimate the concentration of THMs (Figure 7).

### 2.3. Influence of pH on THMs Formation in the Water Disinfection Process

**Figure 8:**
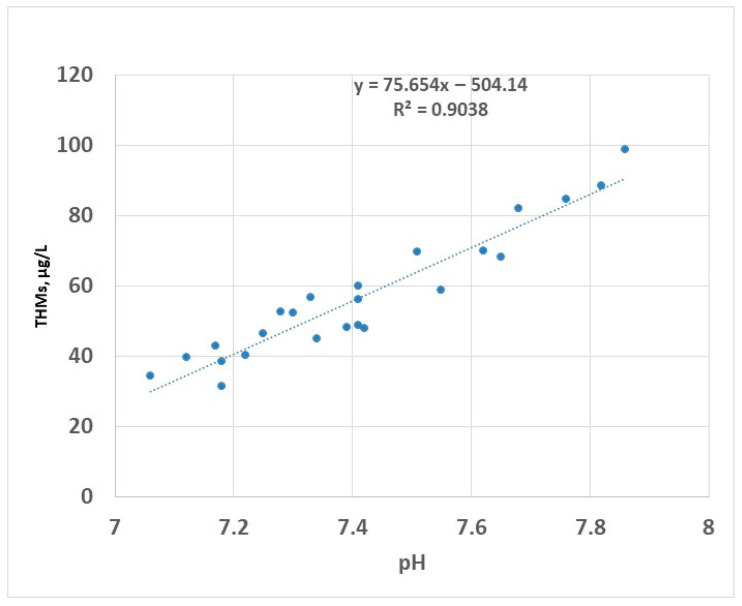
THMs (sampling 2) vs. pH—linear regression.

Chlorine-based disinfectants have been widely used in the water disinfection process. The reaction of chlorine species with organic matter is a concern for two reasons: available chlorine can be consumed by the existing organic compounds, resulting in improper disinfection, and secondly, the formation of the DBPs. Many studies have been conducted to evaluate the role of hypochlorous acid (HOCl) and hypochlorite ion (OCl^−^) in the production of reaction by-products with particular interest in the production of THMs and HAA5. The available form of chlorine in water is dependent on pH: in acidic solutions, the predominant form is HOCl, while at an alkaline pH, the predominant form is OCl^−^ [48]. Of these species, the most active biocidal agent is hypochlorous acid [49,50]. To avoid excessive formation of gaseous chlorine, the pH of chlorinated water is controlled to values between 6.5 and 7.5 using chemical agents (e.g., citric acid) to stabilise the disinfectant (the hypochlorous acid and the hypochlorite ions referred to as free chlorine) [51]. During the two years of testing, the pH of raw water ranged between approximately 7.1 and 7.9 (Figure 8); in this interval, the predominant form of the disinfectant is the most efficient, i.e., HOCl.

The linear correlation between pH and THMs has been previously reported [52]; this is valid for pH values lower than 8 [53]. Disinfection in alkaline conditions (pH higher than 8.5) has been reported to decrease the formation of THMs in the drinking water [25]. This was attributed to the decomposition of organic matter in alkaline environment to the detriment of participation in the process of formation of THMs. However, in practice, the disinfection at an alkaline pH is limited by a reduced efficiency of the process because the active compound HOCl has a lower prevalence in these conditions.

**Figure 9:**
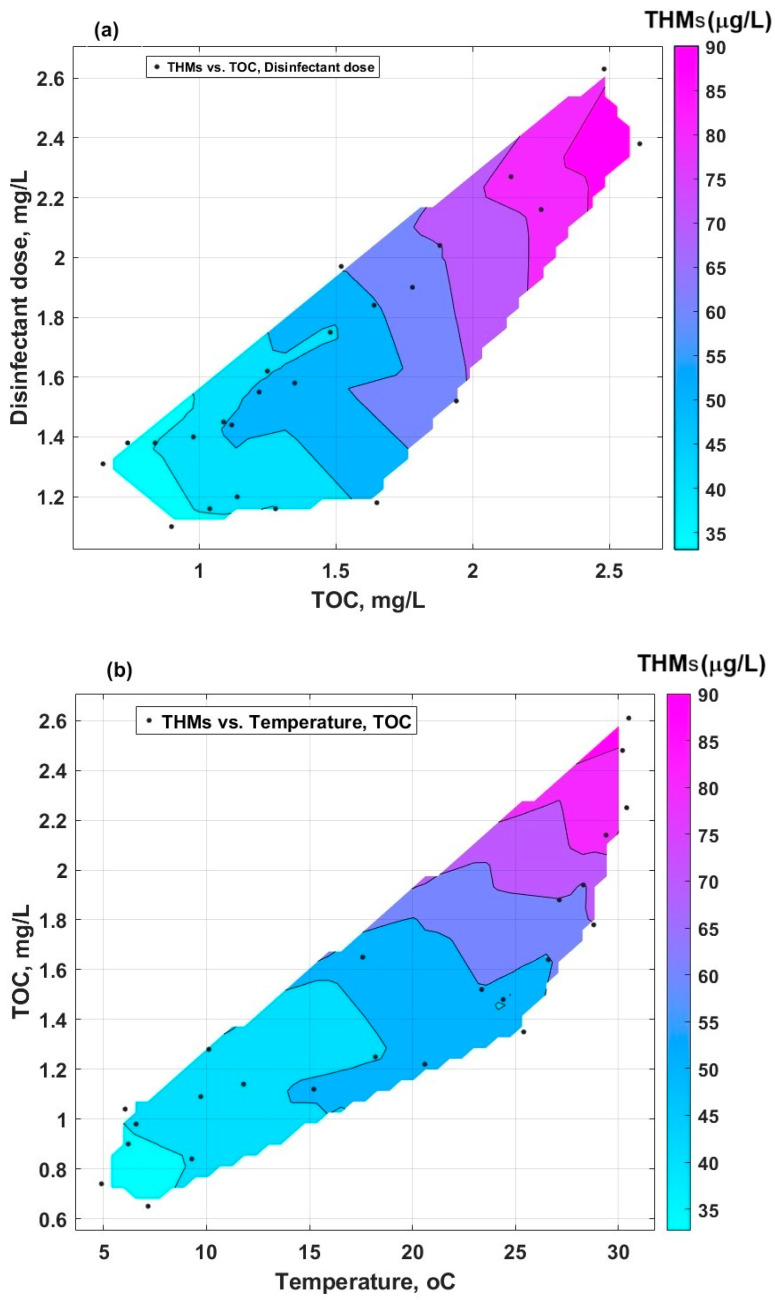
Contour plot of interpolated data: (**a**) THMs vs. TOC and disinfectant dose; (**b**) THMs vs. temperature and TOC.

The representation of the combined influence of two parameters on the formation of THMs by linear data interpolation in MATLAB R2024b software is given in Figure 9. Figure 9a is characterised by a more extensive area with high levels of THMs, compared with Figure 9b. This indicates that the cumulative effect of the disinfectant dose and TOC (Figure 9a) is more important than the effect of the temperature correlated with TOC (Figure 9b). This means that whenever there are organic compounds of humic nature and HOCl, even though the temperature is not that high, the conditions for the formation of THMs are met (the influence of temperature alone on the formation of THMs is low).

With the aim of developing a useful link between the levels of THMs in water and the parameters that influences the formation of THMs (TOC, DIS, T and pH), the statistical method of multiple linear regression (MLR) was used. This method assumes a linear dependence between the level of THMs in water and the independent variables. Multiple linear regression analysis made in MATLAB R2024b gives the following correlation between the content of THMs and the most important parameters:

THMs = 2.2702 + 22.5686 × OC+ 12.7646 × DIS + 0.0330 × T(1)

DIS—disinfectant dose, mg/L;

T—temperature, °C.

**Figure 10:**
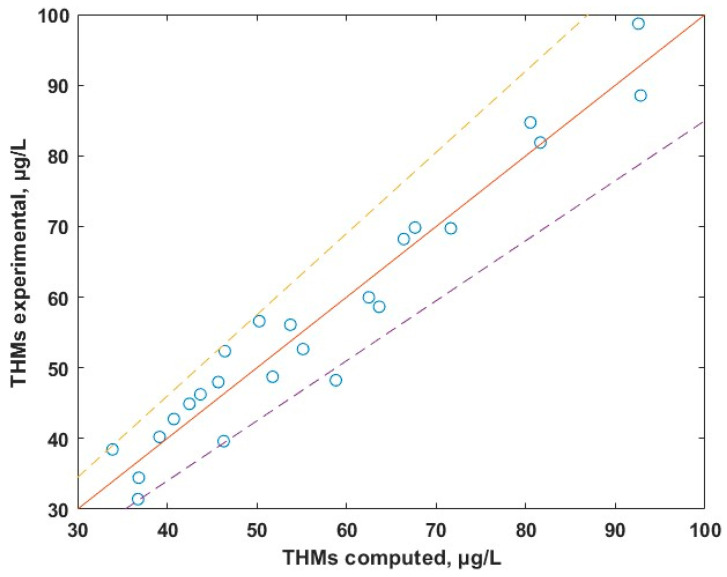
Experimental vs. calculated concentrations of THMs.

The experimental data versus calculated data dependence is given in Figure 10 and shows a good agreement between theoretical and experimental data (the points are uniformly distributed across the ideal red line). Equation (1) predicts the experimental data with an average relative deviation below 10%. Results of MLR suggests that TOC is the major parameter that dictates the levels of THMs. The temperature influences indirectly the levels of THMs by its contribution to the biomass development within the water. The contributing factor of the pH, being of the order 10^−4^, is negligible.

## 3. Materials and Methods

**Figure 11:**
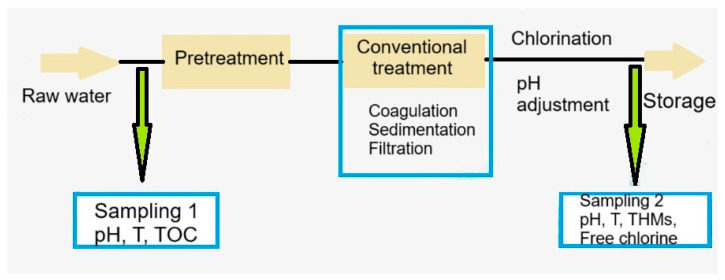
Flow chart of the water treatment processes and the location of sampling points.

The sampling was conducted before the pretreatment stage and after chlorination step (Figure 11). The samples were collected for two years, in three climatic periods: cold and low precipitations (December–February), mild temperatures and heavy rainfall (March, April, October, and November), and warm and dry (May to September). At the time of the sampling, three samples for each location were collected, so that the experiments could be performed in triplicate. The standard deviations are less than 10% of the experimental average.

The differences in TOC content between point 1 of sampling and before chlorination point can be attributed to a partial elimination of organic matter in the water treatment plant. However, as also has been highlighted by previous studies, the elimination of organic compounds is not advanced if more efficient technologies are not available (e.g., pre-ozonation, use of membrane bioreactors) [28,54,55].

Water was collected in 50 mL glass vials filled to the maximum capacity to prevent volatilisation of organic substances. Water samples collected for each month were stored at 4 °C and tested within 24 h of collection.

### 3.1. Materials

Chemical reagents were purchased from Merck/Sigma-Aldrich Chemical (Darmstadt, Germany) THMs used for chromatograph calibration (99% minimum purity), sodium hypochlorite (10% active chlorine), methanol (HPLC grade, 99.9%), ethanol (HPLC grade, 99.8%), sulphuric acid (98%), soluble starch, iodine (99.8 for titration), sodium thiosulfate (0.1 N, titripur), methyl orange (ACS reagent 85%), aluminium sulphate (98%) and isooctane (ACS reagent). Ultrapure water was produced by the Milli-Q Integral system (Merck, Bucharest, Romania).

### 3.2. Analysis of THMs

Water was analysed from two sampling locations: sampling point 1 gave the TOC concentration in the raw water, while sampling point 2 gave the content of THMs formed in the chlorination step. The natural water samples collected from the Arges River (sampling point 1) were subjected to the disinfection process with sodium hypochlorite in the laboratory. The optimal amount of sodium hypochlorite that would lead to maximum disinfection efficiency (i.e., complete removal of pathogens from the water—Escherichia Coli, coliform bacteria) was experimentally determined [56,57]. The procedure consisted of the following steps: 200 mL of natural surface water to be disinfected was introduced into six Berzelius beakers; different amounts of sodium hypochlorite were added (to achieve chlorine concentrations between 1 and 2.8 mg/L); the samples were shaken for 20 min; after that, they were filtered and the amount of formed THMs was analysed.

THMs in drinking water were extracted with isooctane and determined chromatographically using an electron capture detector, according to Romanian standard STAS 12997-91 [58]. A total of 6 mL of water sample was placed in contact with 1 mL of isooctane in a container that was shaken for 10 min (using TOC X5 Shaker device from Hach, Bucharest, Romania), the container was left to stand to allow the phase to separate. The THMs extracted in isooctane were analysed with a Shimadzu 2010 GC equipped with AOC 5000 autosampler (Shimadzu, Bucharest, Romania). The amount of THMs formed under different conditions of TOC, temperature, and pH was determined using the above-mentioned method.

### 3.3. Total Organic Carbon (TOC)

TOC was determined using the method described in EN 1484:1997 [59]. The water samples were diluted and homogenised before being analysed. The TOC determination was performed in three steps: (1) the sample was added to the reaction tube containing acid, then the bottle was placed in the TOC X5 Shaker device (Hach, Bucharest, Romania) to release the inorganic carbon of the sample (total inorganic carbon—TIC); (2) after removing the TIC, the reaction bottle was attached to the pH indicator bottle via a double-sided cap with a gas-permeable membrane; (3) the flask was then placed in the DRB200 digestion system (DRB—digital reactor block, Hach, Bucharest, Romania) for 2 h at 100 °C; the analysis was performed using the DR6000 UV-VIS Spectrophotometer (Hach, Bucharest, Romania) with RFID technology (radio frequency identification).

### 3.4. Free Chlorine

The term “free chlorine” refers to the combination of Cl_2_, HOCl, and OCl that are present in water and are available for water disinfection [60]. This is determined according to the method described in STAS 6364-78 [61].

### 3.5. pH Determination

The pH was determined according to the standard SR ISO 10523:1997 [62]. The pH and temperature were measured using a portable meter HQ40D (HACH, Bucharest, Romania).

## 4. Conclusions

This paper presents an exploratory study that highlights the main factors leading to peak concentrations of THMs in drinking water. The samples were analysed for two years and over three climatic periods: cold and low precipitations (December–February), mild temperatures and heavy rainfall (March, April, October and November), and warm and dry (May to September).

The formation of trihalomethanes in drinking water is dependent on the concentration and nature of organic material (expressed as TOC) in the raw water, the disinfectant dose used in the treatment process, the pH, and the temperature. The concentrations of THMs are directly proportional to the concentration of organic compounds, temperature, and disinfectant dose. Optimal control of all these parameters is necessary to minimise the formation of secondary compounds. The quality of the raw water is very important; the main mechanism used to prevent the formation of trihalomethanes and other disinfection by-products is the removal of organic matter by advanced pretreatment of the water before disinfection. Otherwise, TOC is not considerably reduced in the water treatment process (when using only coagulation and filtration), therefore the risk of the formation of THMs is maintained.

The experimental results obtained indicated that for 2022 the highest amount of THMs was 98.7 mg/L corresponding to the highest temperature registered for that year, namely 30.5 °C (in July). In 2023, at the highest temperature point registered in the experiments, 30.4 °C, a value of 81.9 mg/L THMs was found. Although the rainfall period increases the turbidity of water sources, the major development of humic organic compounds is directly linked to the water temperature. The maximum value of THMs was linked with the maximum value of TOC, temperature, a pH higher than 7.5, and disinfectant dose higher than 2 mg/L. The values of the THMs agree with the European normative but local exceeding points can be registered if the water distribution system is charged with organic deposits on the inner wall surface. In the context of global warming, another factor that can lead to exceeding the limit for THMs is the temperature; higher temperatures mean an increased level of humic compounds in the water source.

According to the graphical interpretations, the pH plays an important role in determining the amounts of by-products formed, namely, low pH values indicate the formation of fewer trihalomethanes. However, the main parameters that are decisive in the formation of THMs are the availability of organic matter and the existence of excess disinfectant.

## Figures and Tables

**Table 1:** Surface water characterisation.

Parameter (Sampling 1)	Minimum Value	Maximum Value
Temperature, °C	4.9	30.5
pH	7.18	7.72
Conductivity, µS·cm^−1^	384	731
Turbidity, NTU	55	278

## Data Availability

Data is contained within the article.
